# COVID-19 diagnosis in a patient with critical limb ischemia: complications and clinical outcomes

**DOI:** 10.1590/1677-5449.200071

**Published:** 2020-09-21

**Authors:** Rafael de Athayde Soares, Rafael Salem Vedovello, Samanta Christine Guedes de Medeiros, Celso Zaffani Nunes, Carlos Alberto Sian, Paulo Daenekas de Melo Jorge

**Affiliations:** 1 Hospital Regional Sul, Serviço de Cirurgia Vascular e Endovascular, São Paulo, SP, Brasil.

**Keywords:** COVID-19, critical limb ischemia, endovascular surgery, angioplasty, peripheral occlusive arterial disease, gangrene, COVID-19, isquemia crítica, tratamento endovascular, angioplastia, doença arterial obstrutiva periférica, gangrena

## Abstract

A 67-year-old male diabetic patient with systemic arterial hypertension was admitted to the emergency department with a necrotic ulcer in the left external malleolus and no palpable popliteal or pedal pulses. Arterial Duplex ultrasound identified femoropopliteal occlusion, with popliteal refilling below the knee and a patent peroneal artery. An endovascular procedure was performed, requiring retrograde access to the popliteal artery to re-establish blood flow and deploy a popliteal stent. Technical success was achieved and the patient underwent debridement of the wound. Two days later, about 48 hours after the operation, the patient began to exhibit respiratory symptoms, with coughing and dyspnea. He immediately underwent a chest CT that identified ground glass opacities, the crazy-paving pattern, and bilateral air bronchogram in the lungs. A reverse transcription – polymerase chain reaction (RT-PCR) test was positive for SARS-Cov-2. The patient was moved to an intensive care unit and put on mechanical ventilation. Both hydroxychloroquine and azithromycin were administered. Despite appropriate treatment, the patient died 4 days after he was diagnosed with COVID-19.

## Resumo

Paciente do sexo masculino, 67 anos, diabético, hipertenso, foi admitido no pronto-socorro com histórico de úlcera necrótica no maléolo externo esquerdo, sem pulsos poplíteos e distais palpáveis. A ultrassonografia Doppler arterial identificou oclusão femoropoplítea, com reenchimento da artéria poplítea infragenicular e perviedade da artéria fibular. Foi realizado procedimento endovascular, com necessidade de acesso retrógrado na artéria poplítea para restabelecer o fluxo sanguíneo e realizar implante de stent poplíteo. O sucesso técnico foi alcançado e, em seguida, o paciente foi submetido ao desbridamento da ferida. No segundo dia, cerca de 48 horas de pós-operatório, o paciente apresentou quadro respiratório com tosse e dispneia. Foi submetido a uma tomografia computadorizada do tórax, que identificou opacidades em vidro fosco e broncograma aéreo bilateralmente nos pulmões, com teste de reverse transcription – polymerase chain reaction (RT-PCR) positivo para SARS-Cov-2. O paciente foi transferido para uma unidade de terapia intensiva, necessitando de ventilação mecânica. Recebeu hidroxicloroquina e azitromicina. Apesar do tratamento em suporte intensivo, o paciente morreu 4 dias após o diagnóstico de COVID-19.

## INTRODUCTION

Covid-19 may disproportionately affect people with cardiovascular disease.^1^^,^^2^ Case series have reported cardiac arrhythmias and cardiac arrest as terminal events in patients with COVID-19.^3^ Moreover, SARS-CoV-2, the causative agent of Covid-19, has been shown to establish itself in the host by exploiting angiotensin-converting enzyme 2 as its cellular receptor.^4^ It is also known that diabetes can increase the risks of infections, including influenza and pneumonia. Indeed, diabetes was present in 42.3% of 26 fatalities due to COVID-19 in Wuhan, China.^5^ Another study in 150 patients (68 deaths and 82 recovered patients) in Wuhan showed that the number of co-morbidities is a significant predictor of mortality.^1^

Notwithstanding the COVID-19 pandemic, critical limb ischemia (CLI) continues to be a life-threatening condition requiring appropriate intervention to avoid mortality and limb loss. The main objective of this paper is to report the case of a patient with CLI who underwent endovascular therapy and then developed a SARS-Cov-2 infection.

### Case report

**Figure 1:**
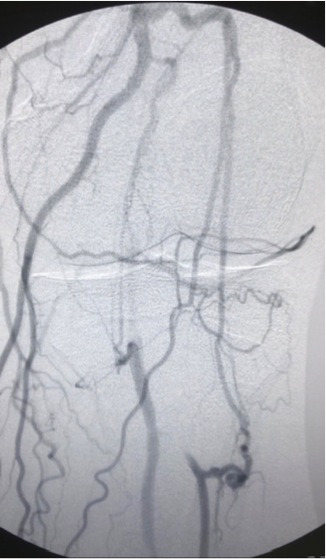
Arteriography showing superficial femoral artery occlusion in Hunter’s canal, with popliteal refilling below the knee and patent peroneal artery.

**Figure 2:**
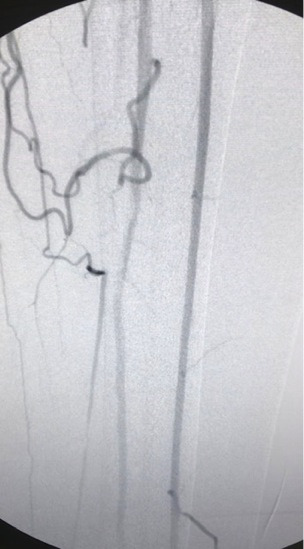
Arteriography showing superficial femoral artery occlusion in Hunter’s canal, with popliteal refilling below the knee and patent peroneal artery.

We report the case of a 67-year-old male patient, with diabetes and taking metformin, active smoker, and with systemic arterial hypertension and taking the angiotensin-converting enzyme inhibitor captopril. He was admitted to an emergency department with an ulcer on the left external malleolus, classified as Rutherford stage 5, with necrosis, foul odor, and no popliteal or pedal pulses palpable. The patient did not report any previous cardiovascular disease. His ankle-brachial index was 0.35. He was examined with Duplex arterial ultrasound of the left limb, identifying superficial femoral artery occlusion in Hunter’s canal, with popliteal refilling below the knee and a patent peroneal artery. Laboratory test results revealed leukocytosis (18 × 10^9^ cells/L) and elevated C-reactive protein (25mg/dl), but the results of renal function tests were normal (creatinine 1.0 mg/dl and urea 25 mg/dl). Given the urgency and the high risk of loss of the limb, the patient underwent endovascular surgery, despite the ongoing Covid-19 outbreak. Spinal anesthesia and sedation were administered to the patient. The procedure was initiated via an anterograde ipsilateral puncture in the common femoral artery, using a 6Fr introducer sheath. Arteriography confirmed the duplex arterial ultrasound findings (Figure 1 and Figure 2).

**Figure 3:**
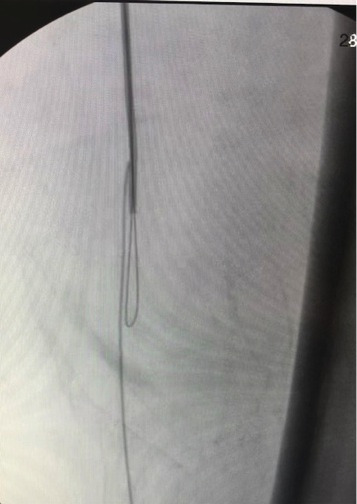
The retrograde guidewire was recovered with a homemade snare, coupled to a 5Fr vertebral catheter and a loop of V18 0.018” 300cm guidewire.

**Figure 4:**
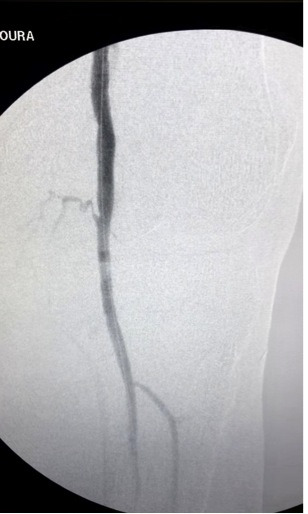
Post-procedure arteriography showing successful artery recanalization.

The patient was then heparinized intravenously with non-fractionated heparin 5000 UI in bolus. A 0.035” 260cm guidewire (Roadrunner® Cook medical) along a 5Fr vertebral catheter (Cook medical) was used to attempt to cross the arterial lesions in the anterograde direction. These attempts to cross the arterial lesion in the anterograde direction failed. Due to this unsuccessful attempt, we decided to perform a retrograde puncture of the popliteal artery below the knee with a 4Fr micropuncture introducer sheath (Cook medical). Successful artery recanalization was achieved with a 0.018” 300cm V18 guidewire (Boston scientific). The retrograde guidewire was recovered using a homemade snare, coupled to a 5Fr vertebral catheter and a loop of a V18 0.018” 300cm guidewire. Anterograde ballooning was performed with a 5×100mm balloon-catheter (armada 18, Abbott). Due to dissection after two ballooning attempts, a self-expandable 6×80 mm stent was deployed (astron pulsar stent). After this, ballooning was performed at the infrapopliteal artery with a 4x60mm balloon-catheter in order to stop the bleeding after removal of the guidewire. The post-procedure arteriography showed successful artery recanalization. (Figure 3 and Figure 4). The ulcer was debrided and dressed appropriately and the femoral introducer sheath was removed from the patient with 15 minutes of manual compression. The patient was then transferred to the ward. The popliteal pulse was present and the ankle brachial-index was measured at 0.97.

**Figure 5:**
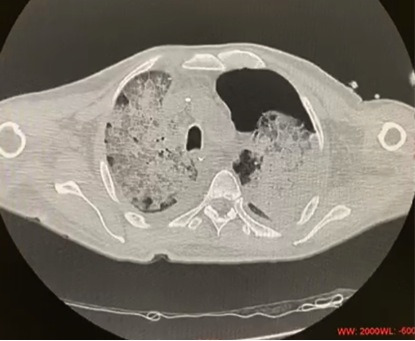
Chest CT scan showing ground glass opacities and the crazy-paving pattern in the lungs.

**Figure 6:**
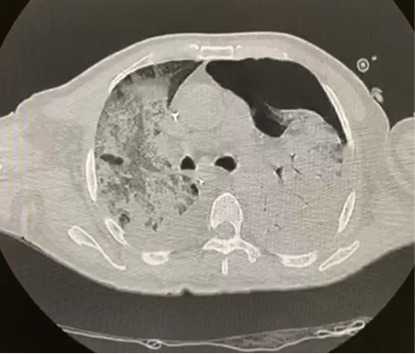
Chest CT scan showing bilateral air bronchogram in the lungs.

Two days later, 48 hours after the procedure, the patient developed respiratory symptoms with coughing and dyspnea. His oxygen saturation was 90% without oxygen supplementation. After clinical stabilization and oxygen supplementation through high flow nasal cannulas, the patient was examined with chest CT that identified ground glass opacities, the crazy-paving pattern, and bilaterally air bronchogram in the lungs (Figure 5 and Figure 6). In view of the Covid-19 pandemic and the high probability chest CT findings, we performed a RT-PCR test, which was positive for SARS-Cov-2. The patient was moved to an intensive care unit and needed mechanical ventilation via orotracheal intubation. Both hydroxychloroquine and azithromycin were administered, in addition to endovenous non-fractioned heparin anticoagulation, because his D-dimer level was greater than 1000mg/dl (3549mg/dl). Another laboratory finding was lymphopenia (0.5 x 10^9^ cells/L). The patient developed renal dysfunction, needing hemodialysis and vasoactive drugs to maintain blood pressure stability. The patient died 6 days after the operation in the intensive care unit because of multiple organ failure, caused by Sars-Cov-2 complications.

## DISCUSSION

The severity of the course of CLI in patients with peripheral arterial occlusive disease is well established in the literature, mainly with regard to poor overall survival and high mortality rates.^6^^,^^7^ The impact of Covid-19 infection on these patients has not yet been thoroughly studied in the literature. In the main, recommendations to avoid elective surgeries are being followed in medical practice, but patients will still need urgent or emergency intervention operations for time-sensitive disease processes such as malignant cancer, critical limb ischemia with high risk of infection and limb loss, or for true emergencies such as traumatic injuries.^8^ Another important factor that can mean that vascular patients with peripheral arterial disease are more susceptible to Covid-19 infection is that they tend to be older patients with medical comorbidities, such as hypertension and diabetes, which are related to poor prognosis in patients infected with Covid-19.^4^^,^^5^ The patient described in this case report was at very high risk of limb loss, which justified the urgent revascularization surgery to which the patient was subjected. Although the results of this procedure were successful, the patient developed respiratory complications postoperatively that needed further intensive critical care and unfortunately caused the patient to die, due to a Sars-Cov2 infection.

Thrombotic complications are emerging as an important issue in patients with COVID-19. Patients infected by this disease are at risk of developing disseminated intravascular coagulation.^9^ Increased levels of D-dimer and fibrin degradation products and prolonged prothrombin time have been associated with poor prognosis in patients affected by the novel coronavirus.^10^ This patient presented with high D-dimer levels, so he was put on full anticoagulation with intravenous non-fractionated heparin. Furthermore, the renal dysfunction was a severe complication that made it necessary to institute hemodialysis in order to maintain clinical stability. A recent meta-analysis by Ali et al.^11^ showed that mortality is significantly higher in patients with COVID-19 with severe acute kidney injury (AKI). AKI stage III occurred in 14/701 (2%) of the patients and was associated with an increased risk of in-hospital mortality (hazard ratio = 9.81, 95% CI:5.46-17.65). Among all the other complications that affected this patient, based on the available limited published data, severe AKI in patients with COVID-19 is an ominous clinical predictor and is associated with high mortality.

Another laboratory finding in this patient was lymphopenia. According to Azkur et al.,^12^ many aspects of severe patients are unique to COVID-19 and are rarely observed in other respiratory viral infections, such as severe lymphopenia and eosinopenia, extensive pneumonia and lung tissue damage, a cytokine storm leading to acute respiratory distress syndrome, and multiple organ failure. This lymphopenia is associated with a defect in antiviral and immune regulatory immunity. At the same time, a cytokine storm leads to extensive activation of cytokine-secreting cells with innate and adaptive immune mechanisms both of which contribute to poor prognosis. This case report describes a patient that had all the clinical and laboratorial findings that contribute to worse clinical course and, unfortunately, caused the patient’s death.

The patient underwent pharmacological treatment with hydroxychloroquine and azithromycin, in addition to full intensive care unit support. No ideal treatment has yet been established in the literature for Covid-19 infection, involving a novel pneumonia caused by a previously unknown pathogen, that emerged in Wuhan, China, and causes SARS-CoV-2 infection. Hydroxychloroquine inhibits pH-dependent steps of the replication of several viruses and has already been quite extensively tested in vitro and in vivo on several different virus strains: African swine fever virus, HIV, SARS-CoV, Influenza A, Chikungunya, Ebola, Zika, and, recently, on SARS-CoV-2.^13^^-^16 In the United States, the Food and Drug Administration issued an Emergency use Authorization on March 30, 2020, that permitted use of these drugs in patients with Covid-19 who were not enrolled on clinical trials. Guidelines suggested that these drugs could be administered to hospitalized patients who had evidence of pneumonia.^17^ However, a recent study published by Geleris et al.^18^ with 1446 patients showed that hydroxychloroquine administration was not associated with either a greatly lowered or an increased risk of the composite end point of intubation or death. In the main analysis, there was no significant association between hydroxychloroquine use and intubation or death (hazard ratio, 1.04, 95% confidence interval, 0.82 to 1.32). Results were similar in multiple sensitivity analyses. There is a need for more studies and trials of treatments for Covid-19 infection in the literature.

It has been demonstrated that the risk of poor outcome is higher in patients with prolonged prothrombin time, international normalized ratio, increased thrombin time, and elevated D-dimer, which indicates coagulopathy that predisposes patients to thrombotic events.^19^^,^^20^ Middeldorp et al.^21^ reported 15% (95% CI, 9.3-22) and 34% (95% CI, 23-46) incidence of venous thromboembolism at 7 and 14 days, respectively. In patients with intracardiac communication such as patent foramen ovale, this poses a risk for paradoxical thromboembolism. This clinical condition may be related to cerebrovascular disease and acute limb ischemia in patients with Covid-19 infection. In this case report, the patient had prior peripheral arterial occlusive disease and was admitted via the emergency room with critical limb ischemia at high risk of limb loss, and underwent endovascular treatment with recanalization of femoropopliteal and peroneal arteries.^22^ The worsening of the ulcer on the left external malleolus and the severity of the patient’s clinical condition may be related to those thrombotic events. However, no further investigations regarding thromboembolic events were conducted due to multiple organ failure and the severity of the patient’s clinical condition, caused by SARS-Cov-2 complications.

## CONCLUSION

This case report describes a patient with CLI who suffered a fatal outcome of Covid-19 infection during the postoperative period after an endovascular procedure, demonstrating that patients with peripheral arterial occlusive disease are linked to poorer prognosis when infected with SARS-Cov-2, due to medical comorbidities such as advanced age, hypertension, and diabetes. More trials of treatments for Covid-19 are needed in the literature.
